# Epigenetic Regulation of Fibroblasts and Crosstalk between Cardiomyocytes and Non-Myocyte Cells in Cardiac Fibrosis

**DOI:** 10.3390/biom13091382

**Published:** 2023-09-12

**Authors:** Liangyu Chu, Daihan Xie, Dachun Xu

**Affiliations:** Department of Cardiology, Shanghai Tenth People’s Hospital, Tongji University School of Medicine, 315 Yanchang Middle Road, Shanghai 200072, China; 2051259@tongji.edu.cn (L.C.); 2051021@tongji.edu.cn (D.X.)

**Keywords:** cardiac fibrosis, cardiac fibroblast, cardiac function, crosstalk, epigenetics

## Abstract

Epigenetic mechanisms and cell crosstalk have been shown to play important roles in the initiation and progression of cardiac fibrosis. This review article aims to provide a thorough overview of the epigenetic mechanisms involved in fibroblast regulation. During fibrosis, fibroblast epigenetic regulation encompasses a multitude of mechanisms, including DNA methylation, histone acetylation and methylation, and chromatin remodeling. These mechanisms regulate the phenotype of fibroblasts and the extracellular matrix composition by modulating gene expression, thereby orchestrating the progression of cardiac fibrosis. Moreover, cardiac fibrosis disrupts normal cardiac function by imposing myocardial mechanical stress and compromising cardiac electrical conduction. This review article also delves into the intricate crosstalk between cardiomyocytes and non-cardiomyocytes in the heart. A comprehensive understanding of the mechanisms governing epigenetic regulation and cell crosstalk in cardiac fibrosis is critical for the development of effective therapeutic strategies. Further research is warranted to unravel the precise molecular mechanisms underpinning these processes and to identify potential therapeutic targets.

## 1. Introduction

Cardiovascular disease, such as ischemic heart disease and hypertension, remains a leading cause of mortality worldwide [1]. Fibrosis is characterized by alterations in extracellular matrix (ECM) components and the excessive deposition of proteins secreted by cardiac fibroblasts (CFs), which represents a common pathological process in chronic inflammatory diseases [2]. Extensive research has demonstrated that cardiac remodeling, including the development of cardiac fibrosis, is a shared phenomenon in various heart diseases [3]. On one hand, cardiac fibrosis can confer beneficial effects, such as facilitating the repair of fibrotic scars subsequent to myocardial infarction (MI), which is crucial for maintaining the structural integrity of the heart [4]. However, the sustained abnormal activation of CFs can lead to disproportionate ECM deposition [5], resulting in reduced cardiac compliance and mechanical dysfunction of the myocardium [6]. Furthermore, the infiltration of a substantial number of CFs within the heart can disrupt normal electrical conduction, as the deposition of secreted substances creates barriers and reduces the electrical coupling among myocardial cells contributing to arrhythmias [7,8].

In clinical studies, the extent of cardiac fibrosis has been identified as an indicator of unfavorable outcomes [9,10]. Despite the established role of fibrosis in heart failure, there is a dearth of effective pharmacological interventions targeting this pathological process in clinical practice. Notably, studies have highlighted the significant role of epigenetics in the progression of cardiac fibrosis [11,12], suggesting that therapeutic strategies based on epigenetic mechanisms may hold promise. Epigenetics refers to heritable modifications in chromatins that do not involve alterations in the DNA sequence. In the cell nucleus, the nucleosome comprises approximately 146 base pairs of DNA and histone octamers. The chemical modifications of both DNA and histones can either enhance or impede the interaction between transcription factors and regulatory proteins, thereby influencing gene transcription.

The proper functioning of cardiac myocytes relies on the regulation via non-myocytes [13], and in turn, cardiac myocytes can also influence the phenotype of non-myocytes via paracrine signaling or direct contact [14,15]. Intercellular crosstalk plays a crucial role in the process of cardiac fibrosis, as various cell types within the heart interact and contribute to its development.

**Figure 1:**
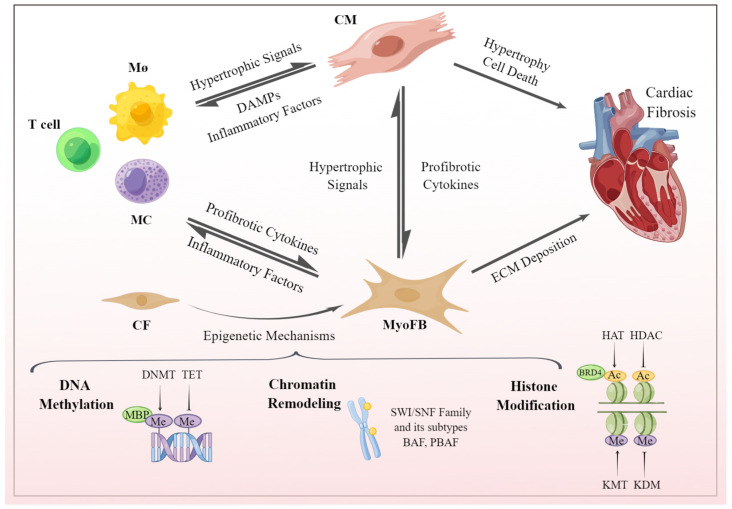
The development of cardiac fibrosis is a multifaceted process. Intercellular signaling and interactions between cardiomyocytes and non-myocyte cells play a crucial role in precisely coordinating various cardiovascular processes. This coordination is achieved via the generation of bioactive factors and the regulation of cytokine secretion. CFs serve as the primary effector cells involved in cardiac fibrosis regulated by epigenetic mechanisms. These two interconnected processes ultimately influence the development of cardiac fibrosis. Ac, acetylation; CF, cardiac fibroblast; CM, cardiomyocyte; Mø, macrophage; MC, mast cell; Me, methylation; MyoFB, myofibroblast.

It is noteworthy that most recent reviews on epigenetics and cardiac fibrosis have primarily focused on DNA, histones, and RNA, with limited attention given to emerging mechanisms such as chromatin remodeling. Our aim is to comprehensively elucidate the epigenetic mechanisms of CFs in cardiac fibrosis, explore the impact of left ventricular fibrosis on cardiac function, and summarize key aspects of intercellular crosstalk between non-myocytes and cardiac myocytes within the heart. Fibroblasts, as crucial effector cells in fibrosis, exhibit intricate epigenetic regulatory mechanisms that contribute to the initiation and progression of cardiac fibrosis. Cardiomyocytes, being the predominant cell type in the heart, play a pivotal role in mediating the interplay between cardiomyocytes and non-cardiomyocytes, thereby influencing the phenotypic transformations in cardiac fibrosis. This review article aims to provide a detailed insight into the epigenetic regulatory mechanisms underlying fibroblasts in cardiac fibrosis while emphasizing the significance of cellular interactions within the cardiac microenvironment (Figure 1). Additionally, we explore the potential of utilizing cardiomyocytes as a bridge to establish the relationship between non-cardiomyocytes and cardiac fibrosis. 

## 2. Epigenetic Regulations of CFs in Cardiac Fibrosis

CFs serve as the primary effector cells in cardiac fibrosis. Under physiological conditions, the proportion of CFs to cardiomyocytes, endothelial cells, and immune cells remains relatively stable [16]. The ECM network, secreted by CFs, plays a crucial role in maintaining the structural integrity of the heart and ensuring normal cardiac conduction [17]. Following acute cardiac damage, such as MI, CFs respond to external signals by activating and expressing genes associated with inflammation and fibrosis [18]. Fu et al., utilizing lineage-tracing models and stage-specific gene profiling, demonstrated that CFs reach their peak activation and proliferation rate within 2–4 days after injury. During this process, they differentiate into myofibroblasts, secrete a substantial ECM, and express α-smooth muscle actin (α-SMA) [19]. Studies have indicated that the activation of fibroblasts and the expression of related genes are regulated via epigenetic mechanisms [20,21]. We aim to review the role of fibroblasts in cardiac fibrosis by focusing on the epigenetic mechanisms.

### 2.1. DNA Methylation

#### 2.1.1. Regulation of DNA Methylation

DNA methylation is one of the most extensively studied epigenetic modifications in mammals. The dynamic changes in DNA methylation pattern involve two processes, de novo DNA methylation and demethylation, which are carried out by a series of enzymes that act as writers, readers, and erasers. Specifically, DNA methyltransferases (DNMTs) act as writers by adding methyl groups to DNA, while methyl-CpG-binding proteins (MBPs) interpret these modifications as readers. During the process of demethylation, several enzymes listed in Table 1, such as ten-eleven translocation (TET) enzymes, act as erasers of epigenetic marks by removing the methyl groups from DNA.

During methylation, the DNA methyltransferase family catalyzes the transfer of a methyl group from S-adenosyl-L-methionine to the fifth carbon of cytosine, resulting in the formation of 5-methylcytosine [22,23]. This modification occurs at sites near cytosine nucleotides or CpG dinucleotides [24], with the majority of these sites located near the promoter and exon regions of genes. DNA methylation reduces the binding of transcription factors to these regions, inhibits the initiation of transcription, and consequently diminishes gene expression. DNMT family members participate in catalyzing methylation as writers. To date, five types of DNMT proteins have been identified, but only DNMT1, DNMT3a, and DNMT3b possess catalytic activity for methyltransferase [25]. DNMT3a and DNMT3b establish new methylation patterns on unmodified DNA, while DNMT1 maintains methylation status by promoting DNA replication and transferring methylation patterns from parent to daughter strands [26,27]. MBP family members act as readers in the methylation process. MBPs contain a methyl-CpG-binding domain (MBD) that specifically binds to CpG positions of methylated DNA, allowing them to enhance DNA methylation and suppress gene transcription [28].

Currently, considerable evidence suggests a close relationship between DNA methylation and fibroblast activation and CFs, as shown in Table 1.

**Table 1:** The roles of DNA methylation modifiers in cardiac fibrosis and CF activation.

Subclass	Modifier	Target	Function	References
DNMTs	DNMT1	SOCS3	Profibrotic, CF activation	[29]
		microRNA-152-3p	Pro-fibrotic, CF activation and proliferation	[30]
		RASSF1A, ERK1/2	Pro-fibrotic, CF proliferation	[31]
	DNMT3a	TRAAK	Pro-fibrotic, CF activation	[32]
		Ras, ERK1/2	Pro-fibrotic, CF activation and proliferation	[33]
		Patched1	Pro-fibrotic, CF proliferation	[34]
		miR-200b	Pro-fibrotic, CF autophagy	[35]
		LncRNA NEAT1	Pro-fibrotic, CF activation	[36]
	DNMT3b	HIF-1α	Pro-fibrotic, CF activation	[37]
		*Rasal1*, *Rassf1*	Pro-fibrotic, CF activation	[38]
		RASSF1A, ERK1/2	Pro-fibrotic, CF proliferation	[31]
MBPs	M2CP2	DUSP5	Pro-fibrotic	[39]
		RASSF1A, ERK1/2	Pro-fibrotic	[40]
		LncRNA GAS5	Pro-fibrotic, CF proliferation	[41]
TETs	TET2	*Hspa1b*	Anti-fibrotic	[42]
		IFN-γ	Anti-fibrotic	[43]
		IL-6	Anti-fibrotic	[44]
	TET3	BMP7	Anti-fibrotic, EndMT	[45]

#### 2.1.2. DNA Methylation in Cardiac Fibrosis

DNMTs. Cardiac fibrosis can occur in various conditions, such as ischemia, volume overload, pressure overload, and hypoxia [46]. Hypoxia is a common pathological process in these factors, and one possible epigenetic mechanism is DNA methylation within cardiac fibroblasts, which causes the inactivation of tumor suppressor gene *Rassf1a* and activates the ERK signaling pathway, resulting in an increase in the number of fibroblasts and ultimately leading to cardiac fibrosis [33,37]. A study by Ayan et al. found that extracellular superoxide dismutase may significantly reduce *Rassf1a* gene methylation and positively regulate the ERK1/2 signaling pathway, thereby alleviating hypoxia-induced cardiac fibrosis [31]. Papait et al. also observed in their study that alterations in the DNA methylation profile of the injured heart can trigger the phenotypic switching of endothelial cells to an interstitial fibroblast-like phenotype, thereby contributing to cardiac fibrosis via the aforementioned pathway [21]. Previous studies have found that hypoxia increases using hypoxia-inducible factor-1α (HIF-1α), mediating the upregulation of DNMT1 and DNMT3b, thus promoting myocardial fibrosis [37]. In their study, He et al. reported that inhibiting the DNMT1-mediated methylation of the *α-sma* promoter DNA has the potential to suppress the differentiation of CFs and prevent fibrosis [47]. Likewise, Tian et al. found that treatment with monocrotaline-induced DNMT1-HIF-1α-PDK-mediated chamber-specific metabolic memory in right ventricular fibroblasts promotes the synthesis of collagen protein and the development of fibrosis [48]. Therefore, DNMT1 represents a promising therapeutic target for anti-fibrotic treatment. However, it is unclear whether DNMT3a plays a role in hypoxia-induced cardiac fibrosis. Recently, a study has shown that by inhibiting the HIF-1α/DNMT3a signaling pathway mediated in the TRAAK channel, the activation of cardiac fibroblasts and the expression of fibrosis-related proteins are significantly reduced, confirming the role of DNMT3a in cardiac fibrosis [49]. In addition, Zhao et al. also found that DNMT3a can regulate the autophagy of fibroblasts and control cardiac fibrosis by controlling the level of miR-200b [35]. 

MBPs. Regarding MBPs, current research has found that methyl-CpG binding protein 2 (MeCP2), as a protein containing MBD, participates in the regulation of cardiac fibroblast proliferation and fibrosis via its ability to bind methylated DNA. In the transverse aortic constriction (TAC) mouse model, inhibiting MeCP2 activates fibroblasts and aggravates cardiac fibrosis [50], while the overexpression of MeCP2 alone shows lower levels of fibrosis and good cardiac repair [51]. Tao et al.’s research on the mechanism of MeCP2 action showed that the use of MeCP2 inhibitors to treat cardiac fibroblasts can increase the expression of dual-specificity phosphatase 5 (DUSP5), and DUSP5 negatively regulates the ERK signaling pathway, thus promoting myocardial fibrosis [39]. Currently, apart from MeCP2, the regulation of other MBPs on CFs and their mechanisms in cardiac fibrosis have not been studied, which may be an important direction for future treatment of cardiac fibrosis.

### 2.2. Histone Modification

#### 2.2.1. Histone Acetylation in Cardiac Fibrosis

The seminal work by Allfrey et al. unveiled the significance of histone acetylation modifications in elucidating the intricate mechanisms governing gene expression [52]. Acetylated histones exert their influence by impeding chromatin condensation, thereby facilitating the access of transcription factors to chromatin and promoting gene expression [53]. Analogous to DNA methylation, histone acetylation necessitates the involvement of writers, readers, and erasers. Among them, histone acetyltransferases (HATs) and histone deacetylases (HDACs) emerge as pivotal enzymes responsible for regulating the extent of histone acetylation [54]. HATs catalyze the transfer of acetyl groups from acetyl-CoA to the N-terminal lysine ε-amino acid group. Michael et al. have categorized these enzymes into five families [55], with particular emphasis on the p300/CBP HATs due to their close association with cardiac fibrosis [56]. Conversely, HDACs generally impede gene expression and have been classified into four families by Annemieke et al.: I, II, and IV encompass zinc-dependent HDACs, while class III necessitates nicotinamide adenine dinucleotide (NAD+) for enzymatic activity [57]. HDAC I and HDAC II are deemed crucial in the intricate mechanisms of myocardial fibrosis [58]. Table 2 provides a comprehensive listing of the main HDACs, their targets, and their functions. Multiple signaling pathways converge upon HATs and HDACs, intricately regulating the expression of fibrosis-related genes and mediating the process of cardiac fibrosis [59].

Histone acetylation modifications are recognized by specific proteins, and Dhalluin et al. have reported the bromodomain-mediated recognition of acetylated Lys residues [66]. Bromodomains (BRDs) exist in various types of nuclear proteins, including HATs, methyltransferases, helicases, and BET protein families [67]. Additionally, the YEATS domain has been identified as another domain with selectivity for binding acetylated histones [68]. Further research holds promise for the discovery and elucidation of additional readers of histone acetylation in the future.

HATs. The acetylation of lysine residues in the histone tail is catalyzed via HATs, which impacts gene expression by inhibiting chromatin aggregation and providing binding sites for proteins with acetylated lysine recognition domains. Extensive studies have focused on p300, revealing its involvement in dysregulated stress signals such as hypertrophy and fibrosis in cardiac cells. It has been observed the recruitment of p300 to genes associated with hypertrophy and fibrosis, as well as the regulation of their expression [69]. This pathway also involves the acetylation of the transcription factor GATA4 and the acetylation of histones H3K9 and H3K27 at the *Gata4* promoter. The activation of endothelin-1 and atrial natriuretic factor promoters in this pathway drives the genomic stress response and contributes to cardiac fibrosis [70]. 

In 2008, Morimoto et al. found that curcumin could disrupt the p300/GATA4 complex in rat cardiac cells, inhibiting cellular hypertrophy induced via agonists and p300 [71]. Curcumin has also demonstrated improvement in the cardiac contractile function in the Dahl salt-sensitive hypertensive rat model and the MI rat model. It reduced collagen deposition and inhibited the proliferation and migration of fibroblasts in the MI mouse [71,72,73]. However, caution is warranted when interpreting these experimental results, considering that curcumin is involved in other pathophysiological processes, such as inflammation and oxidative stress in the heart [74]. The discovery and application of two p300 small-molecule inhibitors, L002 and C646, further confirmed the role of p300 in cardiac fibrosis [75,76]. However, these inhibitors seem to lack a certain degree of selectivity and efficacy [77]. Recently, the development of A485, a highly selective small-molecule inhibitor for p300 and CBP, may provide better insights into the role of HATs in myocardial fibrosis and demonstrate the potential of HATs inhibitors in clinical applications. However, its application in the field of cardiac fibrosis has not been reported yet. CBP30 and CBP112 are small-molecule inhibitors that target the bromodomain of p300/CBP. They prevent HATs from binding correctly to acetylated histone marks on chromatin without affecting the catalytic function of p300/CBP [78,79]. In the future, the discovery of highly selective and potent inhibitors targeting the bromodomain and catalytic domain of HATs will further elucidate the role of HATs in controlling cardiac fibrosis and their potential to block cardiac fibrosis remodeling. 

HDACs. HDACs catalyze the deacetylation of lysine residues on histone proteins, which contrasts the function of HATs. However, emerging evidence suggests HDACs are also involved in the complex network of fibrotic and anti-fibrotic factors in cardiac fibrosis. 

In 2002, HDAC II was shown to inhibit cardiac hypertrophy by suppressing the activity of MEF2 [80]. Since then, the involvement of HDACs in cardiac fibrosis has gained increasing recognition. Studies have demonstrated that class I HDACs can activate fibroblasts and act as regulatory factors in cardiac fibrosis [12]. They likely achieve this by inhibiting the transcription of anti-fibrotic genes [81], mediating pathological processes such as mitochondrial overactivation and calcium overload in human CFs [82], and promoting fibroblast proliferation and migration [83]. Other class I HDACs have also been implicated in the occurrence and development of cardiac fibrosis [84,85,86]. 

HDAC inhibitors (HDACis) have shown a potential to inhibit cardiac fibrosis by targeting the function of HDACs. For instance, the representative compound vorinostat/suberoylanilide hydroxamic acid has demonstrated the ability to inhibit cardiac fibrosis in preclinical models such as MI and TAC [87]. It received FDA approval in 2006 for the treatment of cutaneous T-cell lymphoma. Another compound, ITF2357/givinostat, has shown improvement in cardiac diastolic function in the murine models of hypertension- or aging-induced diastolic dysfunction with preserved ejection fraction, with no significant fibrosis observed [88]. However, hidden fibrosis was detected in this model using quantitative mass spectrometry and atomic force microscopy, but it was still inhibited by ITF2357/givinostat [89], indicating that the compound effectively halted the progression of early cardiac fibrosis. In recent years, selective HDACis have been discovered, offering a promising approach for precise intervention in histone acetylation modifications. For example, RGFP966, a selective HDAC3 inhibitor, has demonstrated significant efficacy in improving cardiomyocyte hypertrophy and interstitial fibrosis in a mouse model of diabetic cardiomyopathy through the DUSP5-ERK1/2 pathway, while also reducing oxidative stress in the heart [84]. Krüppel-like factor 4 (KLF4) is a zinc finger-containing transcription factor that regulates cell growth and differentiation [90]. The inhibition of class I HDAC with SK-7041 leads to histone acetylation in the promoter region of *Klf4*, resulting in the upregulation of its expression and the suppression of *Nppa* promoter activity, counteracting the cell size increase induced via norepinephrine [91]. In a study conducted by Patel et al., the selective inhibition of HDAC2 using sodium butyrate in a rat model of cardiac hypertrophy induced by partial abdominal aorta constriction (PAAC) resulted in a reduction in collagen levels between myocardial cells and an enhancement in mitochondrial DNA concentration, although the underlying pathway was not proposed [92]. Notably, Jebessa et al. revealed that the abhydrolase domain containing 5 (ABHD5) acts as a serine protease, cleaving HDAC4 and generating an N-terminal peptide (HDAC4-NT) both in vitro and in vivo. The gene delivery of *Hdac4-nt*, using AAV9 as a vector, improved cardiac hypertrophy and fibrosis without altering the level of cardiac lipid deposition. The transfer of *Hdac4-nt* not only normalized the expression of *NPPB* but also influenced the expression levels of the downstream *Mef2* gene, *Xirp2*, and nerve growth factor IB (*Nr4a1*). These findings suggest that the ABHD5-mediated expression of *Hdac4-nt* is sufficient to inhibit the activation of the classical fibrotic pathway induced by *Mef2*, unveiling the potential of endogenous cardiac fibrosis inhibitors targeting epigenetic regulation [93].

Despite the promising findings, the clinical approval of these compounds for the treatment of cardiac fibrosis is currently unavailable due to their potentially unknown molecular mechanisms. The role of different HDACs in the process of cardiac fibrosis is not consistent [94], which introduces certain risks when utilizing pan-HDACi for the treatment of cardiac fibrosis treatment. Furthermore, the targets of HDACs beyond histones in cellular contexts remain incompletely elucidated, and the use of pan-HDAC inhibitors may result in lethal cytotoxicity by inhibiting key HDACs. Additionally, the development of specific inhibitors that selectively target different cells and types of HDACs is challenging due to the shared structural domains among class I, II, and IV HDACs [94].

As a key enzyme in epigenetic regulation, the impact of HDACs on cardiac fibrosis has consistently been a matter of great concern. Therefore, it is imperative to acquire a more comprehensive understanding of the functions and targets associated with different classes of HDACs. Consequently, the design of drugs that specifically target various cells and types of HDACs becomes essential.

Bromodomain Extraterminal Protein. Bromodomains are extensively studied proteins involved in recognizing ε-N-acetylated lysine motifs using their BRDs, which serve as protein interaction modules [95]. Among them, BRD4, a member of the BET family, has been implicated in the activation of CFs, myocardial hypertrophy, and cardiac fibrosis [96]. BRD4 facilitates binding to acetylated histones via its BD1 and BD2 BRDs and activates RNA polymerase II to initiate gene transcription via its carboxy-terminal domain in a complex with a positive elongation transcription factor [97]. Additionally, BRD4 contributes to the formation of dynamic cell state-specific enhancers, called super-enhancers (SEs). In activated fibroblasts, the TGF-β signaling pathway induces BRD4 to bind to enhancers in a p38-dependent manner [98]. For instance, BRD4 binds to an enhancer element located 65 kb downstream of the *Meox1* gene, which encodes a homeobox transcription factor, forming a SE. The SE then loops back to the *Meox1* promoter, promoting *Meox1* expression and initiating a cascade of pro-fibrotic gene expression [99]. Moreover, BRD4 promotes pro-fibrotic gene expression in cardiomyocytes by forming SEs and facilitating fibroblast activation via cell–cell contact or paracrine signaling, ultimately leading to fibrotic remodeling [100].

Researchers have discovered that JQ1, an acetyl-lysine mimic, can displace BRD4 from chromatin binding in cardiac fibrosis. In the mouse models of TAC and *Plnr9c* mutation-induced dilated cardiomyopathy (DCM), JQ1 has demonstrated the ability to improve cardiac contractile dysfunction, reduce cardiomyocyte hypertrophy, and attenuate cardiac fibrosis [101]. Moreover, JQ1 inhibits the expression of *Meox1* induced by SEs in CFs [99]. Clinical studies of apabetalone, a selective inhibitor of the BD2 domain of BRD4, have shown that the treatment group experienced a reduced number of hospitalizations for heart failure associated with type 2 diabetes and recent acute coronary syndrome compared to the control group. Furthermore, apabetalone exhibited good tolerability, suggesting that BRD4 may serve as a potential effective therapeutic target for cardiac fibrosis [102]. 

However, recent studies have provided evidence suggesting the involvement of BRD4 in maintaining the homeostasis of mitochondrial function in cardiomyocytes, thereby preserving normal cardiac function [103]. These findings underscore the importance of further investigating the functions of BET proteins and the molecular mechanisms regulated by BET inhibitors. Such investigations are crucial for enhancing drug-targeting strategies and mitigating potential unknown risks associated with BET inhibition in the context of cardiac fibrosis.

#### 2.2.2. Histone Methylation in Cardiac Fibrosis

In addition to acetylation, histone methylation has emerged as another histone modification associated with cardiac fibrosis. Histone methylation is catalyzed via histone methyltransferases (HMTs), which include lysine methyltransferases (KMTs) and arginine methyltransferases (RMTs), depending on the specific site of modification. Lysine demethylases (KDMs) can reverse this modification [104]. The impact of histone methylation on gene expression is site specific, where the methylation of H3K4, H3K36, and H3K79 is typically linked to transcriptional activation, while the methylation of H3K9 and H3K27 leads to transcriptional repression [105].

Over 90% of KMTs possess a SET (su(var)3–9, an enhancer of zeste, and trithorax) domain. Among them, the EZH2 (an enhancer of zeste homolog 2) family, which includes a SET domain, has been identified to regulate H3K27 methylation in cardiac fibrosis [106,107]. Studies have revealed the elevated EZH2 expression in the atrial myocytes and fibroblasts of patients with atrial fibrillation, accompanied by atrial fibrosis and enhanced differentiation of the atrial fibroblasts. The inhibition of EZH2 using the inhibitor GSK126 has been shown to attenuate Ang-II-induced atrial enlargement and fibrosis [108]. Another study demonstrated that EZH2 mediates the H3K27 methylation of the *Mir-30d* promoter, leading to the suppression of miR-30d expression and contributing to pathological cardiac hypertrophy [109]. Ge et al. discovered that the lncRNA NEAT1 recruits EZH2 to the *Smad7* promoter region via physical binding, resulting in the suppression of *Smad7* expression and the exacerbation of cardiac fibrosis progression [110]. Furthermore, Yuan et al. reported that the inhibition of the function of Wdr5, which catalyzes the trimethylation of lysine 4 on histone H3, could induce cell cycle arrest in CFs and alleviate cardiac fibrosis by activating the Mdm2/p53/p21 pathway [111].

Currently, KDM is primarily categorized into two subfamilies: lysine-specific demethylases (LSDs) and JMJC domain-containing family (JMJD) [104]. Within the LSD1/KDM1 subfamily, a pivotal role in cardiac fibrosis is attributed to the regulation of H3K4 and H3K9 methylation. In a rat model of TAC, cardiac fibrosis was mitigated via muscle-specific *LSD1* knockout, resulting in the inhibition of the TGF-β signaling pathway and the attenuation of systolic dysfunction, cardiac hypertrophy, and fibrosis [112]. KDM3A, another member of this subfamily, can bind to the *Timp1* promoter and enhance its transcription. TIMP1, a marker of cardiac fibrosis, can activate CFs and induce fibrosis, which can be counteracted by the pan-KDM inhibitor JIB-04 [113]. Notably, our recent study unveiled a significant reduction in JMJD4 levels in patients with DCM. Augmenting the abundance of JMJD4 can uphold the metabolic homeostasis of myocardial cells and mitigate cardiac fibrosis by reducing histone methylation. JMJD4 holds promise as a novel target for future therapeutic interventions in heart diseases and bears significant clinical translational value [114].

The precise role and impact of histone methylation in cardiac fibrosis remain inadequately understood. Therefore, conducting more comprehensive investigations focusing on specific subtypes of enzymes is imperative to unravel the intricate involvement of histone methylation in cardiac fibrosis.

### 2.3. Chromatin Remodeling

#### 2.3.1. Regulation of Chromatin Remodeling

Chromatin remodeling refers to the molecular mechanisms that alter the packaging state of chromatin, including histones in nucleosomes and corresponding DNA molecules during processes such as gene expression, replication, and recombination [115]. A large portion of the genome is inaccessible, which necessitates chromatin remodeling for gene expression, and this process is closely related to the function of chromatin remodeling complexes [116]. Chromatin remodeling complexes aid in constructing the initial state of chromatin and use ATP hydrolysis energy to catalyze the transition of chromatin structure to alternative states, making DNA regulatory sequences accessible to transcriptional machinery. Meanwhile, mediating the activation or inhibition of target gene transcription by transcription factors [117]. 

#### 2.3.2. Chromatin Remodeling in Cardiac Fibrosis

Currently, chromatin remodeling complexes are divided into four major families based on their primary sequence and ATPase subunit structure: the SWI/SNF family, the imitation SWI family, the chromodomain-helicase-DNA-binding family, and the INO80 complex family [117,118]. Among these, only the SWI/SNF complex family has been clearly reported to be closely related to cardiac fibrosis.

SWI/SNF. The SWI/SNF chromatin remodeling complex was first discovered in yeast cells [119]. However, its high-resolution structure had not been reported until 2019 when cryo-electron microscopy was used to reveal the high-resolution structures of the yeast SWI/SNF complex family and human BAF/PBAF complexes [120,121,122]. The research progress on the specific mechanisms by which SWI/SNF affects chromatin remodeling has also accelerated. With further research, its subtypes BAF (BRG1-associated factors) and PBAF (polybromo-associated BRG1-associated factors) have been confirmed to be associated with cardiac fibrosis.

BAF. BAF can actively participate in the process of cardiac fibrosis by affecting ubiquitination modification. In mice with cardiac-specific *Baf155* knockout treated with Ang II, the Ang II-induced heart dysfunction, cardiac hypertrophy, and fibrosis were significantly reduced, while the overexpression of *Baf155* showed mild cardiac hypertrophy and aggravated vascular thickening and fibrosis induced by Ang II [123]. In addition, Zhang N et al. found that BAF155 regulates the downstream target poly (ADP-ribose) ylation (PARylation) by controlling PARP1 ubiquitination and degradation, affecting the process of cardiac fibrosis [123]. Similarly, in mice with *Wwp2* knockout, the level of PARP1 ubiquitination modification in the heart was significantly reduced, which could promote cardiac fibrosis by enhancing downstream target PARylation [124]. Therefore, *Baf155* may modulate cardiac fibrosis by controlling the ubiquitination level of PARP1 and regulating the level of downstream targets’ PARylation. Sun et al. found that the intrinsic deficiency of BAF60C may regulate the processes related to cardiac fibrosis by affecting the MEF2/SRF co-factor myocardin target [125]. Additionally, BAF57, BAF180, BAF200, and others have all been shown to be associated with cardiac remodeling and fibrosis [126,127,128]. In summary, the BAF chromatin remodeling complex is closely related to myocardial fibrosis, and BAF155 may become a potential therapeutic target for the future treatment of cardiac fibrosis.

PBAF. The mechanism of action of PBAF in cardiac fibrosis is not yet clear, but several studies have reported a close association between PBAF and cardiac fibrosis. Zhou et al. found that KDM2B interacts with *Brg1* to promote the chromatin accessibility of the *Il-6* promoter via non-canonical functions independent of its demethylase activity [129]. BRG1 can also activate the stress overload-induced 3β pathway by inhibiting *Hdac2*, affecting cardiac hypertrophy and fibrosis [130]. In addition, *Brg1* defects can prevent the aggregation of neutrophils around endothelial cells [131], and *Brg1* has also been shown to play an important role in cardiac embryonic development [132]. These effects of *Brg1* may have an impact on cardiac fibrosis. In the future, as research on PBAF deepens, it may provide different perspectives for the treatment of cardiac fibrosis.

## 3. The Impact of Cardiac Fibrosis on Cardiac Function

Cardiac fibrosis refers to the changes in the quantity and quality of the interstitial myocardial collagen network produced via the heart in response to ischemic injury, systemic diseases, drugs, and other harmful stimuli that affect the cardiovascular system or the heart itself. Previous studies have shown that cardiac fibrosis can alter the structure of the myocardium, leading to an impaired contraction and relaxation function of the heart [133]. Additionally, cardiac fibrosis can induce arrhythmias and contribute to the development of heart failure [134]. Overall, the impact of fibrosis on cardiac function is predominantly negative and can result in decreased myocardial contraction and relaxation functions, arrhythmias, and conduction block, altered cardiac structure and morphology, and disrupted cardiac metabolic function, among other issues. Therefore, investigating the specific mechanisms by which fibrosis affects cardiac function is significant for the treatment and prevention of cardiovascular diseases.

The left ventricle is the primary pump of the heart, responsible for pumping oxygenated blood into the aorta for distribution to the body while returning lower PaCO_2_ blood to the lungs for oxygenation. Previous studies have shown that the left ventricular function significantly affects the right ventricular contraction, with approximately 20% to 40% of the right ventricular systolic pressure and volume output derived from the left ventricular contraction [135]. Therefore, the left ventricular function is critical for maintaining the metabolic needs of various tissues and organs in the body. Existing evidence suggests that the degree of cardiac fibrosis in human patients and various cardiac disease models is closely associated with adverse outcomes in the left ventricle. Therefore, investigating left ventricular fibrosis is currently an important direction for studying the impact of cardiac fibrosis.

Currently, most studies indicate that left ventricular fibrosis has a negative impact on contractile function via several mechanisms. Firstly, it may disrupt the coordinated action of excitation–contraction coupling in the myocardium, thereby affecting excitation–contraction coupling [136]. Secondly, fibrosis can interfere with myocardial cell perfusion by inducing microvascular dysfunction, leading to inadequate oxygen and nutrient supply to the myocardium and impairing left ventricular contraction function [136,137]. Thirdly, collagen deposition in fibrotic areas can activate proteinase-dependent pathways, leading to the degradation of original fibrous collagen and destruction of the linkage between sarcomere contractile units and the ECM, resulting in impaired left ventricular contraction function [136]. Fourthly, certain types of fibrosis may lead to an increased secretion of mediators that inhibit cardiac contraction function by activating immune cells in the cardiac interstitium [138,139]. Therefore, understanding the mechanisms by which fibrosis affects left ventricular function is crucial for developing effective treatments for cardiovascular diseases.

The current prevailing view is that left ventricular relaxation function impairment is mainly caused by deposition of perimysial and intramyocardial fibrous tissue [140], which increases the degree of cross-linking between the original fibrous components and leads to increased left ventricular stiffness [141,142]. Echegaray et al. evaluated the myocardial collagen volume fraction and Young’s modulus of type I and III collagen in 40 patients with preserved ejection fraction and heart failure symptoms. They found a significant positive correlation between the content of type I collagen and the stiffness of the ECM, as well as the severity of left ventricular relaxation function impairment [143].

In addition to impairing left ventricular contraction and relaxation function, fibrosis can also interfere with left ventricular electrical signal conduction, leading to severe arrhythmias. The current theory is that the effect of fibrosis on left ventricular conduction function is mainly due to the interaction between CFs generated by fibrosis and neighboring cardiac cells after establishing gap junction connections. This interaction generates pro-arrhythmic electrical stimuli [144,145]. Rubart et al. found in a mouse MI model that when fibroblasts electrically coupled with border zone cardiomyocytes, the voltage ECM in low cell-density area, leads to the interruption of left ventricular impulse conduction and impaired left ventricular conduction function [146]. Therefore, understanding the impact of fibrosis on left ventricular electrical signal conduction is essential for developing effective treatments for arrhythmias associated with cardiovascular diseases.

## 4. Crosstalk between Myocytes and Non-Myocyte Cells in Cardiac Fibrosis

Cardiac fibrosis is a complex physiological process involving various stages of cell transcriptional expression, proliferation, differentiation, and morphological changes [147]. Among these stages, the intercellular signaling and tissue interactions between myocytes and non-myocyte cells play a critical role in precisely coordinating diverse cardiovascular processes. This coordination is achieved via the regulation of cytokine secretion and bioactive factor production, ultimately influencing the development of cardiac fibrosis. Extensive research has been dedicated to understanding the crosstalk between fibroblasts and cardiomyocytes, revealing numerous cytokines and signaling pathways associated with cardiac fibrosis [148,149]. Moreover, literature reports have highlighted the significance of crosstalk between T cells, macrophages, mast cells, and cardiomyocytes in influencing the occurrence of cardiac fibrosis [150,151,152]. Investigating the process of intercellular crosstalk between myocytes and non-myocyte cells is crucial for enhancing our understanding of the underlying mechanisms of cardiac fibrosis and may yield novel therapeutic targets. In this section, we will primarily focus on discussing the importance of crosstalk between T cells, mast cells, macrophages, fibroblasts, and finally, cardiomyocytes.

### 4.1. Crosstalk between CFs and Cardiomyocytes

As the predominant cell types in the heart [153], CFs and cardiomyocytes play crucial roles in cardiac physiology and pathology. In end-stage chronic heart disease, cardiac fibrosis often coexists with heart failure [140], indicating the presence of communication and interaction between cardiomyocytes and CFs. Understanding the crosstalk between these two cell types can shed light on the relationship between cardiac dysfunction and fibrosis, and harnessing their interaction may impede the progression of chronic heart disease.

On one hand, under stress conditions, cardiomyocytes can activate fibroblasts via phenotypic transformation [154,155]. Conversely, activated CFs synthesize excessive collagen fibers that deposit in the ECM, thereby influencing the phenotype of cardiomyocytes and the mechanical microenvironment they inhabit [156]. The impact of fibrosis in the left ventricle on cardiac function has been discussed in Section 3 of this review. Additionally, CFs and cardiomyocytes can sense mechanical stress via mechanosensitive channels, integrins, calcium-related signaling, and other pathways, leading to changes in gene expression and cellular remodeling [157]. Current research suggests that these two types of cells can interact via paracrine signaling (including exosomes), modifications in the ECM, and metabolic regulation.

#### 4.1.1. CFs Alter the Phenotype of Cardiomyocytes

CFs engage in communication with cardiomyocytes via the secretion of cytokines such as IL-1, IL-6, TNF-α, TGF-β, and IGF-1 [158]. This regulatory mechanism may have both beneficial and detrimental effects. For example, IL-11 secreted by CFs can lead to cardiomyocyte dysfunction and ventricular injury [159]. Conversely, IL-33, also secreted by CFs, can mitigate myocardial hypertrophy induced by Ang II and adrenaline, as well as pressure overload-induced myocardial fibrosis [160]. CFs are also capable of secreting Ang II [161], which is associated with left ventricular reactive hypertrophy, reduced coronary blood flow reserve, interstitial fibrosis, decreased capillary density in the heart [162], and pro-arrhythmic effects [163]. Fibroblast growth factor-2, predominantly secreted by CFs, not only acts on receptors on the surface of cardiomyocytes to promote hypertrophy but also stimulates CFs to secrete additional cytokines via autocrine signaling [164].

Paracrine signaling mediated via exosomes derived from CFs establishes communication with cardiomyocytes. Bang et al. discovered that CF-secreted miR-21-3p induces cardiomyocyte hypertrophy by inhibiting Sorbin and SH3 domain-containing protein 2 and PDZ and LIM domain 5, and pharmacological inhibition can reverse this pathological process [165]. Similarly, miR-27a is believed to cause cardiomyocyte hypertrophy through the same pathway [166]. In a study on myocardial ischemia-reperfusion injury, CFs upregulated miR-423-3p, which increased the survival rate of cardiomyocytes via exosomes [167].

CFs are recognized as the primary source of ECM proteins, including collagen, elastin, and fibronectin, and they transmit signals via integrin receptors on their cell surface [168]. In 1994, Fisher et al. demonstrated the crucial role of collagen in maintaining the differentiated phenotype of cardiomyocytes in the ECM [169]. Therefore, the ECM can be considered an intermediary between CFs and cardiomyocytes. Under conditions of altered load, the normal balance of ECM collagen subtypes in the heart is disrupted, leading to an increase in the proportion of type I collagen [170]. Creemers et al. observed that mice deficient in TIMP-1 exhibited increased matrix metalloproteinase (MMP) activity and more severe myocardial hypertrophy in a mouse model of MI, resulting in a significant loss of interstitial collagen fibers [171]. Recent studies have shown that MMP9, an enzyme that extensively degrades ECM [172], regulates cardiomyocyte autophagy, and inhibiting MMP9 increases autophagic flux, thereby preventing congestive heart failure after MI [173]. ECM deposition is often considered detrimental to cardiac function. Interestingly, Russo et al. discovered that activated CFs with TGF-β/Smad3 activation can preserve the ECM network by inhibiting matrix-degrading proteases in a TAC mouse model, thereby reducing damage to cardiomyocytes [174]. Notably, our research team has found that the inhibition of fibroblast activation protein (FAP) leads to increased peri-infarct vascularization, promoting ECM deposition and the arrangement of CFs, which prevents their excessive activation and reduces cardiac fibrosis. Furthermore, through *Fap* knockdown or inhibition in *Nppb* (encoding pre-pro BNP) and *Npr1* (encoding BNP receptor) deficient mice, we observed that the cardioprotective effect of *Fap* inhibition was lost. Inhibiting *Fap* stabilizes BNP to reduce cardiac fibrosis and promote cardiac repair. Targeted drugs related to FAP may become a focal point in the future heart disease treatment [175].

Furthermore, during the differentiation of CFs into myofibroblasts, the nutritional and mechanical conditions of surviving cardiomyocytes may further deteriorate, exacerbating the severity of fibrosis. Wang et al. conducted a study revealing that GSK-3β mediates the activation of NLRP3 inflammasomes and the production of IL-1β in CFs after MI, leading to increased expression of caspase-3 and N-GSDMD in ischemic myocardial cells. This process also results in an elevated Bax/Bcl-2 ratio, thereby exacerbating myocardial apoptosis [176]. Additionally, Zou et al. demonstrated that under conditions of pressure overload, CFs exhibit an increased secretion of Wnt5a or Wnt11, which promotes myocardial apoptosis and fibrosis via the activation of the FZD5 and EGFR signaling pathways [177]. In summary, the intercellular signaling pathways between CFs and cardiomyocytes in the context of fibrosis are diverse and hold potential as research directions for future treatment targets.

#### 4.1.2. Cardiomyocytes in Pathological State Mediate Activation of CFs

Cardiomyocytes have the ability to synthesize cytokines and hormones, which enables them to exert a paracrine influence on the phenotype of neighboring fibroblasts. Transforming growth factor is synthesized and released via cardiomyocytes in response to mechanical stretch, thereby activating fibroblasts [178,179]. Cardiomyocytes also produce leukemia inhibitory factor (LIF), which inhibits the differentiation of CFs into myofibroblasts. This action partially counteracts the pro-fibrotic effect of TGF-β, thus slowing down the progression of cardiac fibrosis and remodeling [180]. Another notable paracrine signal is atrial natriuretic peptide (ANP), secreted by atrial myocytes. ANP affects fibroblast proliferation and the secretion of ECM proteins. In patients with persistent atrial fibrillation, the myocardial levels of ANP were found to be only 1/6 of those in the control group. This decrease was accompanied by the loss of ANP receptors on fibroblast membranes. Restoring ANP levels in these patients may have a beneficial effect on atrial fibrillation [181].

Datta et al. conducted a study demonstrating that cardiomyocyte-derived exosomes containing Hsp90 and IL-6 are transferred to fibroblasts during cardiac hypertrophy, leading to a change in the phenotype of fibroblasts via the activation of the STAT3 pathway [182]. Furthermore, increased levels of Hsp20 within cardiomyocytes have been found to release protective exosomes in diabetic mice, resulting in reduced cardiac hypertrophy and fibrosis [183].

SIRT2, an NAD+-dependent histone deacetylase, plays a crucial role in various physiological processes and heart-related diseases [179]. Tang et al. revealed that overexpression of *Sirt2* specifically in the heart has been shown to alleviate aging-related cardiac dysfunction and reduce Ang II-induced cardiac hypertrophy and fibrosis. These beneficial effects are likely mediated via epigenetic mechanisms [184].

The interaction and underlying mechanisms between CFs and cardiomyocytes are crucial factors in maintaining heart function and understanding the development of heart disease. However, the precise details of this crosstalk remain unclear. As described above, investigating the role of MMP and the ECM network in pathological conditions can provide insights into the crosstalk between these cell types. Such understanding will aid in identifying new targets and developing novel strategies for the prevention and treatment of heart disease. 

### 4.2. Crosstalk between Macrophages and Cardiomyocytes

In the context of cardiac fibrosis, crosstalk between macrophages and cardiomyocytes holds significant importance. Resident macrophages are unevenly distributed in various tissues of the human body, and their specific functions vary depending on the microenvironment of each tissue [185]. Within the heart, resident cardiac macrophages are closely situated near cardiomyocytes, and even direct cell-to-cell contact can occur [15,186]. In the fibrotic heart, macrophages release a range of cytokines and proteins, thereby altering the microenvironment surrounding cardiomyocytes. This spatial arrangement and resulting microenvironmental changes form the basis for structural and functional crosstalk between macrophages and cardiomyocytes. The primary modes of interaction and influence between macrophages and cardiomyocytes in cardiac fibrosis include intercellular communication, matrix remodeling, growth and proliferation, phenotype switching, and mitochondrial dysfunction [15,187,188,189,190]. 

Current studies have revealed that macrophages can influence cardiomyocytes via various paracrine pathways, thereby participating in the occurrence and development of cardiac fibrosis. In the diabetic mouse model, the elevated levels of NLRP3 activate macrophages to secrete IL-1β. This secretion induces a decrease in potassium current and an increase in calcium current in cardiomyocytes by prolonging the action potential duration, ultimately promoting cardiac fibrosis [191]. In addition to simply promoting fibrosis, macrophages can exhibit different fibrotic effects at different stages; in the inflammatory model of the heart, the increased secretion of MMP9 by macrophages enhances the expression of Mer tyrosine kinase in the early stage of inflammation. This process leads to the decomposition of the ECM in the damaged heart and the clearance of dying cardiomyocytes, thereby reducing cardiac fibrosis [192]. However, in the late stage of inflammation, the elevated levels of MMP-9 can promote the transformation of fibroblasts and the deposition of collagen in the myocardial interstitium by upregulating the expression of pro-inflammatory genes, thus increasing cardiac fibrosis [193,194]. Additionally, Zlatanova et al. found that the lack of hepcidin can increase the number of CCR2+ macrophages, which, in turn, release IL-4 and IL-13 by enhancing the expression of phosphorylation activator of STAT3, thereby promoting cardiomyocyte renewal and affecting cardiac fibrosis [195]. Furthermore, macrophages can also influence cardiac fibrosis by impacting the mitochondria of cardiomyocytes. In cardiac fibrosis, the increased presence of macrophages causes mitochondrial damage in cardiomyocytes and triggers the release of danger-associated molecular patterns (DAMPs). These DAMPs can bind to formyl peptide receptor 1 and Toll-like receptors, respectively, activating the inflammatory signaling pathway and leading to the production of various chemokines. Consequently, these chemokines increase the recruitment of neutrophils, mast cells, and other cells, thereby exacerbating the release of inflammatory factors associated with cardiac fibrosis and worsening fibrosis [187,196]. 

Cardiomyocytes have the ability to secrete various cytokines and growth factors through autocrine pathways, which can impact neighboring macrophages and contribute to the occurrence and development of cardiac fibrosis. In their study, Liu et al. found that in the TAC model, MRTF-A could potentially regulate macrophage trafficking and induce cardiac hypertrophy by activating *Itgb2* transcription [197]. In the experimental models of MI and myocardial hypertrophy, the expression of TGF-β secreted by cardiomyocytes is significantly upregulated, which can activate *Smad3* in macrophages. Through the SMAD3-dependent pathway, TGF-β mediates the phenotypic transformation of macrophages, leading to the increased production of anti-inflammatory cytokine IL-10 and VEGF, thereby promoting the differentiation of myofibroblasts and ECM synthesis and accelerating cardiac fibrosis [198,199]. Cardiac myocytes can secrete various secretory factors, which can affect cardiac fibrosis through multiple pathways such as influencing macrophage phenotype switching and enhancing activation. In patients with DCM, the heightened expression of PAI-1 in cardiomyocytes is involved in the polarization of M2 macrophages in the heart. M2 macrophages can enhance the secretion of inflammatory factors associated with cardiac fibrosis. Thus, cardiomyocytes can influence cardiac fibrosis by impacting the polarization of M2 macrophages [190,200]. Additionally, the secretion of VEGF, TNF-α, IL-1β, and other factors via cardiomyocytes can stimulate the activation and cytokine secretion of macrophages, further promoting the progression of cardiac fibrosis [138]. 

In summary, the interaction between macrophages and cardiomyocytes plays a crucial role in the development of cardiac fibrosis. Gaining a deeper understanding of these interactions can aid in the development of novel treatment strategies for cardiac fibrosis. Modulating the interaction between macrophages and cardiomyocytes may offer new therapeutic approaches to prevent the progression of cardiac fibrosis and improve cardiac function, including inhibiting specific cytokines, regulating macrophage polarization, reducing inflammation, promoting intercellular communication, and regulating matrix remodeling processes to restore normal cardiac structure. However, future research is still required to gain more experimental evidence to determine the effectiveness and safety of these interventions.

### 4.3. Crosstalk between T Cells and Cardiomyocytes

As a crucial component of humoral immunity, the role of T cells in cardiac fibrosis has gained recognition. Certain subsets of T cells may directly activate fibroblasts or indirectly stimulate macrophages [6]. The role of regulatory T cells in cardiac fibrosis appears to be contradictory [201,202]. Currently, the precise interaction between T cells and cardiomyocytes remains largely unknown. Rieckmann et al. discovered that the release of myosin heavy chain α by dying cardiomyocytes could potentially trigger the activation of T helper cells, contributing to the pathogenesis of experimental autoimmune myocarditis [203]. Another study revealed that cytotoxic T cells are activated after MI and can recognize and eliminate non-ischemic neonatal cardiomyocytes in vitro [204]. Liao et al. observed that a specific subset of T cells, such as γδT cells, induce cardiomyocyte apoptosis by secreting IL-17A [205]. However, T cells can also exhibit a protective effect on cardiomyocytes. In 2009, a study found that the transfer of CD4+CD25+ regulatory T cells effectively improved cardiac injury and reduced cardiac fibrosis induced by Ang II in hypertensive mice [206]. 

Further investigation is necessary to elucidate the interaction between T cells and cardiomyocytes. With the advent of CAR-T therapy, it may be possible to target specific cells (e.g., fibroblasts) to mitigate the progression of cardiac fibrosis in the near future. However, potential side effects such as cytokine release syndrome should be minimized as much as possible.

### 4.4. Crosstalk between Mast Cells and Cardiomyocytes

The proliferation of mast cells may relate to the development of cardiac fibrosis. Experimental data have demonstrated that mast cell expansion plays a pivotal role in fibrotic pathogenesis by influencing fibroblast transformation and the production of macrophage-related MMPs [207,208,209]. As important constituent cells of the heart, cardiomyocytes also play a critical role in cardiac disease [210]. Therefore, crosstalk between mast cells and cardiomyocytes may represent a significant mechanism underlying cardiac fibrosis, although the direct interaction between the two cell types remains largely unknown. Investigating the mechanisms of interaction between mast cells and cardiomyocytes in cardiac fibrosis may offer new therapeutic targets.

Currently, mast cells can indirectly impact the function of cardiomyocytes via the secretion of various bioactive molecules, contributing to the development of cardiac fibrosis. For instance, mast cells can release TNF during degranulation [211], promoting cardiac fibrosis by inducing cardiomyocyte apoptosis and MMP-9 production [212]. During piecemeal degranulation, mast cells produce IL-1β [213], which affects cardiomyocytes in a similar manner to TNF, thereby promoting cardiac fibrosis remodeling [214]. Wang et al. discovered that blocking TNF and IL-1ß can reduce subsequent fibrosis remodeling and cardiomyocyte apoptosis in a model of hypertension-induced heart disease [215]. While a substantial body of evidence suggests that mediators derived from mast cells contribute to fibrosis by impacting cardiomyocytes, promoting the generation of fibroblasts, and facilitating collagen deposition in the interstitium, there are also experimental studies indicating that mast cell-derived mediators can counteract the development of cardiac fibrosis by indirectly influencing the survival or expression profile of growth factors in cardiomyocytes [216]. 

In summary, the crosstalk between mast cells and cardiomyocytes in cardiac fibrosis does not necessarily promote the development of fibrosis, and this effect may vary depending on the microenvironment changes. With further investigations into this mechanism, it might be possible to target specific pathways for the treatment of cardiac fibrosis. However, it is crucial to avoid potential side effects, such as an excessive secretion of systemic inflammatory factors secretion, during this process.

## 5. Conclusions and Prospects

Epigenetic regulation and cell crosstalk play crucial roles in the initiation and progression of cardiac fibrosis. Although multiple regulatory mechanisms have been identified in the process of cardiac fibrosis, the interplay between these mechanisms remains elusive. Moreover, JMJD4 and BNP exhibit significant clinical translational value and may serve as critical therapeutic targets in future heart disease, deserving consideration in clinical practice. Future research should delve into the specific effects of epigenetic regulatory mechanisms in cardiac fibrosis, extending beyond CFs alone. However, the current technological limitations pose challenges in investigating the precise epigenetic behavior of individual cell types. When implementing interventions on specific cell types under “co-culture” conditions, there is a high likelihood of inducing phenotype and state changes in other cells, thereby influencing the outcomes of epigenetic studies. Similarly, achieving a precise targeting of cells and molecules in animal and clinical experiments has always been a challenging issue, raising concerns about the accuracy of data derived from animal studies and clinical research. Nevertheless, with the advancement of research techniques and the discovery of new biomarkers, we anticipate that these challenges can eventually be overcome.

Additionally, it is essential to elucidate the crosstalk between different cell types within the heart. Epigenetic regulation and cell crosstalk offer potential avenues for targeted therapies at the molecular, cellular, and organ levels. However, careful attention must be paid to the potential toxic side effects of these drugs on the heart and other organs, while also striving to enhance drug efficacy by minimizing these side effects. Additionally, due to individual variations, the relative contributions of different mechanisms to cardiac fibrosis may vary among individuals. Therefore, personalized and precise treatment approaches should be pursued, utilizing techniques such as gene sequencing and other relevant methods.

## Figures and Tables

**Table 2:** The roles of histone deacetylation modifiers in cardiac fibrosis [60,61,62,63,64,65].

Class	Modifier	Target	Function
I	HDAC 1	Histone, ATM, p53	Pro-fibrotic, CF activation, and myocardial hypertrophy
	HDAC 2	Histone, GATA, α-SMA	Pro-fibrotic, CF activation, and myocardial hypertrophy
	HDAC 3	Histone, GATA, STAT3, FOXP3	Pro-fibrotic, collagen accumulation, and myocardial hypertrophy
	HDAC 8	p38-MAPK, Hsp70, SMC3	Pro-fibrotic, cell proliferation
IIa	HDAC 4	N.A. ^1^ (weak catalytic activity, may act as scaffolding proteins)	Pro-fibrotic, hypertrophy
	HDAC 5		
	HDAC 7		
	HDAC 9		
IIb	HDAC 6	α-tubulin, Ku70, cortactin, tau	Cell mobility, repair of protein misfolding, and pro-fibrotic
	HDAC 10	Histone, Akt, Hsp70	DNA repair, autophagy, and immunoregulation
III	Sirtuins 1–7	Including but not limited to histone, SOD2, cytochrome c	Mainly in DNA repair and oxidative stress
IV	HDAC 11	BRD2, NLRP3	Immunoregulation, DNA replication

^1^ N.A., not applicable.
